# Anterolateral papillary muscle rupture caused by myocardial infarction: A case report

**DOI:** 10.1186/1757-1626-1-172

**Published:** 2008-09-20

**Authors:** Suriya Jayawardena, Anne S Renteria, Olga Burzyantseva, Gowda Lokesh, Louis Thelusmond

**Affiliations:** 1 Coney Island Hospital, 2601 Ocean Parkway, Brooklyn, NY 11235, USA

## Abstract

**Background:**

The rupture of the anterolateral papillary muscle is less common than the posteromedial papillary muscle since the anterolateral muscle has dual blood supplies, while the posteromedial papillary muscle has a single blood supply.

**Case presentation:**

We present a case report of a 42 year old male presenting with heart failure being diagnosed to have mitral regurgitation from the partial rupture of the anterolateral papillary muscle due to coronary artery disease. The patient underwent a mitral valve replacement and concomitant coronary artery bypass grafting of the first and the second obtuse marginal arteries.

**Conclusion:**

Acute mitral regurgitation can be precipitated by acute myocardial infarction due to rupture of the anterolateral papillary muscle.

## Background

Rupture of a papillary muscle is an uncommon but often fatal complication of acute myocardial infarction (MI) which is responsible for approximately 5% of death after MI [1]. The characteristics of the underlying coronary disease will define the clinical presentation and prognosis, the mortality could be as high as 80% during the first week of post MI [1]. The rupture of the posteromedial papillary muscle is most common seen in about 75% of cases. Posteromedial has a single blood supply from the posterior descending branch of a dominant right coronary artery, and is associated with inferior wall infarctions. The rupture of the anterolateral muscle is less common, occurring in 25% of cases, as it has dual blood supplies: from the first obtuse marginal, originating from the left circumflex; and from the first diagonal branch, originating from the left anterior descending. The rupture of the latter is seen with anterolateral MI [2-4]. Papillary muscle rupture is usually seen in relatively small area infarctions, often with modest coronary disease extent revealed by angiogram [1].

## Case report

A 42-year-old Asian man presented to the emergency room with progressively worsening dyspnea over the past five days, now at minimal exertion and occasionally also at rest, associated with productive cough with pinkish sputum for the same period of time. He complained of chest tightness, diaphoresis, orthopnea, paroxysmal nocturnal dyspnea, and decreased effort tolerance for over the same period of time. Past medical history was remarkable for hypertension, hyperlipidemia for ten years, non-compliant with medications, active smoker, and Family history of coronary artery disease, father died of myocardial infarction at the age of fifty-two.

**Figure 1:**
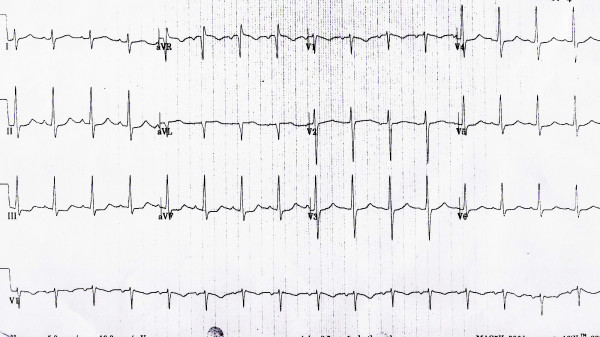
EKG showing normal sinus rhythm, HR 100/min, normal axis and ST segment depression in the II/III/aVF and V_5–6 _(Inferolateral Ischemia).

On physical exam patient was in respiratory distress sitting up right, blood pressure was 150/90 mmHg, heart rate 100 bpm, regular, respiratory rate 28/min. Neck veins were not distended and he had no ankle edema. On examination of the cardiovascular system he had a regular S1 S2 with a S4 gallop. No murmur was appreciated. He had bilateral coarse crepitation all over the lungs. Initial EKG showed ST depression at inferolateral leads (fig 1). The chest radiography showed cardiomegaly with congestive heart failure.

**Figure 2:**
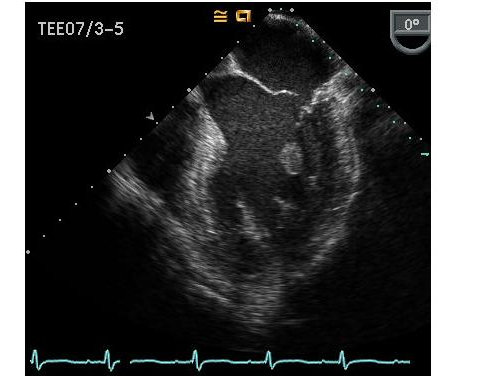
Transesophageal Echocardiogram showing an echo density in the left ventricle suggestive of partial ruptured anterolateral papillary muscle.

**Figure 3:**
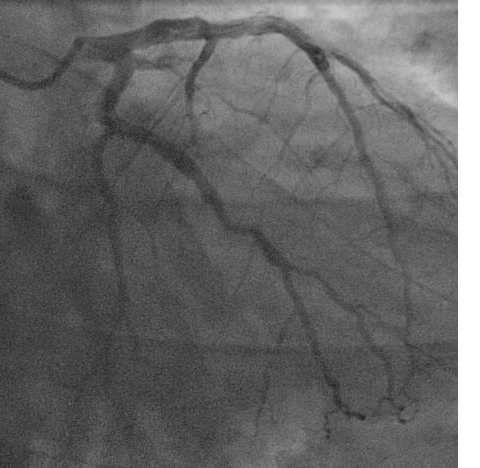
Coronary angiogram showing a non dominant circumflex with a small caliber first obtuse marginal (OM1) totally occluded at the proximal end and a large caliber second obtuse marginal with a 75% stenosis in the mid portion.

The patient was admitted to the coronary care unit with the diagnosis of decompensated heart failure secondary to acute coronary syndrome (CK 294/308/426, Troponin 0.126/0.122/0.105), and treated accordingly. A transthoracic echocardiogram was performed, revealing a normal left ventricular ejection fraction (LVEF) and moderate to severe mitral regurgitation (MR). A transesophageal echocardiogram was performed subsequently which showed that the mitral valve was normal in thickness with a flail anterior leaflet not coapting with the posterior leaflet. Color flow Doppler revealed severe MR. Effective Regurgitant Orifice (ERO) was 5 mm^2^, regurgitant volume by PISA was 60 ml, regurgitation fraction was 60%, and the Vena Contracta width was 8 mm. There was a holosystolic flow reversal of the pulmonary vein. Echo density visualized in the left ventricle suggested partial ruptured anterolateral papillary muscle (fig 2). Left ventricle was hyperdynamic, no wall motion abnormalities were seen and the LVEF was more than 65%. The diagnosis of severe MR with preserved left ventricular function was made. Subsequent coronary angiogram revealed a non dominant circumflex with a small caliber first obtuse marginal (OM1) totally occluded at the proximal end and a large caliber second obtuse marginal with a 75% stenosis in the mid portion (fig 3). The other coronary arteries had wall irregularities but no stenosis, and the right coronary artery was dominant.

**Figure 4:**
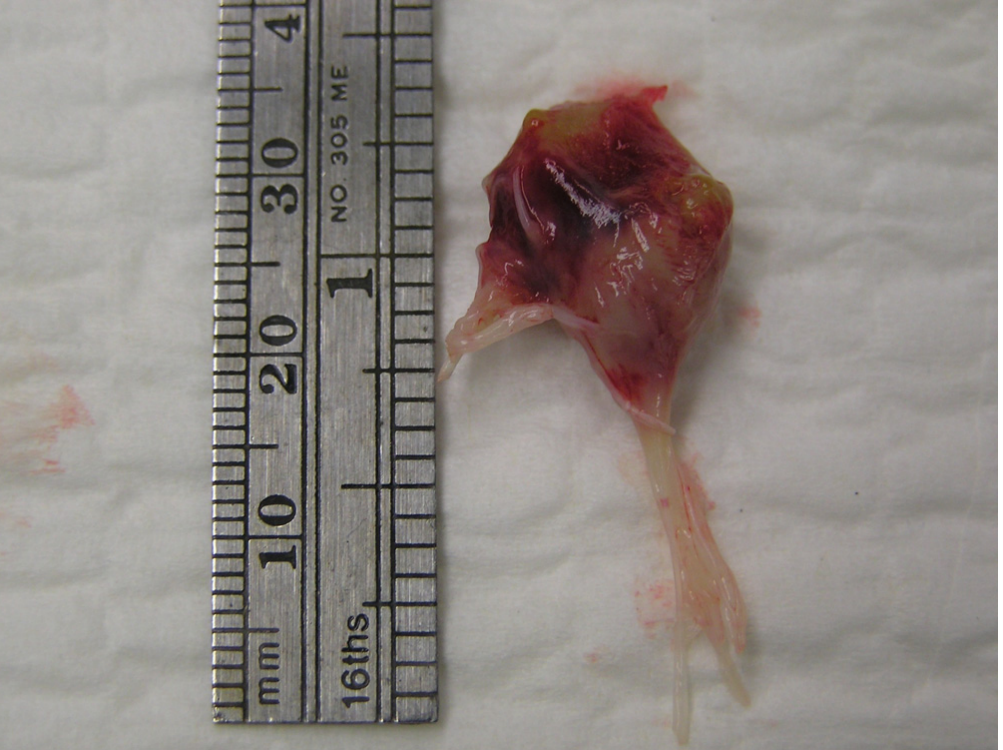
Specimen of the resected anterolateral papillary muscle.

The patient underwent a mitral valve replacement with a St Jude mechanical valve and concomitant coronary artery bypass grafting of the first and the second obtuse marginal arteries (fig 4). He had an uneventful recovery, and was discharged with minimal dyspnea on exertion. He was then seen in the follow up clinic two weeks after the surgical intervention, patient was asymptomatic and with a class II heart failure (NYHA). The transthoracic echocardiogram performed in the clinic showed a normal LVEF, mild flattening of the IV septum – a common finding after a valve replacement – and a functional mechanical mitral valve with no paravalvular leaks.

## Discussion

We presented the case of a patient admitted status post-acute myocardial infarction secondary to the occlusion of the first obtuse marginal, with consequent MR as a mechanical complication of the MI and congestive heart failure. When the transesophageal echocardiogram was performed the MR was found to be secondary to the partial rupture of the anterolateral papillary muscle, which could not be appreciated with a transthoracic echocardiogram.

The clinical presentation and severity of a papillary muscle rupture depends on the involved coronary artery and Left ventricular performance. This is usually clinically apparent 6 days post-acute MI [1], compatible with the presentation of our patient. As stated previously, the anterolateral papillary muscle is less often involved in a rupture than the posterior papillary muscle, because of its dual blood supply.

Different types of lesions to the papillary muscle may occur as a complication of ischemia; prolapse, elongation or rupture in different degrees, partial rupture being the most common type of rupture [5]. The precise diagnosis of papillary muscle rupture can be difficult to establish by transthoracic echocardiography, as the ruptured head may not prolapse into the left atrium, making transesophageal echocardiography a more sensitive and useful tool for diagnosis [6-8].

Due to the high mortality rates with the medical management of papillary muscle rupture impose urgent surgical intervention, the timing of intervention being dictated by the patient's hemodynamic stability [9-12]. The survival rates seem to be related to the extent of papillary muscle rupture, with the best results occurring when a small portion of the tip is ruptured, related to small infarction and limited coronary disease [13].

## Conclusion

This case confirms the importance of an immediate echocardiographic evaluation in establishing the diagnosis, whenever an acute mechanical complication from an acute MI is suspected. The definitive therapy is surgical valve repair [14] or most often, replacement, which should be undertaken as soon as possible because clinical deterioration in these patients can be sudden.

## Abbreviations

MI: Myocardial Infraction; CK: Creatinine kinase; LVEF: Left Ventricular Ejection Fraction; M: Mitral Regurgitation; ERO: Effective Regurgitatant Volume; PISA: Proximal Isovelocity Surface Area: OM1: Obtuse Marginal Artery 1; OM2: Obtuse Marginal Artery 2; NYHA: New York Heart Association; IV: Interventricular Septum.

## Authors' contributions

SJ, AR and OB treated the patent and was responsible for writing the paper and looking up the back ground references. GL was responsible for proof reading and cross checking the references. LT was responsible for over all coordination and final proof reading. All the above mentioned authors read and approved the final manuscript.

## Consent

A written informed consent was obtained from the patient for publication of this case report and accompanying images. A copy of the written consent will be made available on request.
